# Supervised Learning With Perceptual Similarity for Multimodal Gene Expression Registration of a Mouse Brain Atlas

**DOI:** 10.3389/fninf.2021.691918

**Published:** 2021-07-28

**Authors:** Jan Krepl, Francesco Casalegno, Emilie Delattre, Csaba Erö, Huanxiang Lu, Daniel Keller, Dimitri Rodarie, Henry Markram, Felix Schürmann

**Affiliations:** Blue Brain Project, Ecole polytechnique fédérale de Lausanne, Genève, Switzerland

**Keywords:** multimodal image registration, perceptual similarity, gene expression brain atlas, Allen mouse brain atlas, non-rigid, machine learning, deep learning

## Abstract

The acquisition of high quality maps of gene expression in the rodent brain is of fundamental importance to the neuroscience community. The generation of such datasets relies on registering individual gene expression images to a reference volume, a task encumbered by the diversity of staining techniques employed, and by deformations and artifacts in the soft tissue. Recently, deep learning models have garnered particular interest as a viable alternative to traditional intensity-based algorithms for image registration. In this work, we propose a supervised learning model for general multimodal 2D registration tasks, trained with a perceptual similarity loss on a dataset labeled by a human expert and augmented by synthetic local deformations. We demonstrate the results of our approach on the Allen Mouse Brain Atlas (AMBA), comprising whole brain Nissl and gene expression stains. We show that our framework and design of the loss function result in accurate and smooth predictions. Our model is able to generalize to unseen gene expressions and coronal sections, outperforming traditional intensity-based approaches in aligning complex brain structures.

## 1. Introduction

Mouse brain atlases are an essential tool used by neuroscientists to investigate relationships between structural and functional properties of different brain regions. The Allen Institute for Brain Science has produced a reference whole brain atlas, associated Nissl stains, and about 20,000 different gene expression atlases obtained using high-throughput *in situ* hybridization (ISH) techniques (Lein et al., 2007; Dong, 2008).

In order to utilize the information provided by the different markers, gene expressions must be aligned to the reference Nissl atlas, so that all the data can be put into a common coordinate system. To this end, the Allen Mouse Brain Atlas (AMBA) includes an alignment module, but this module is limited to non-deformable transformations (Sunkin et al., 2012). For this reason, previous works (Erö et al., 2018) have had to resort to a manual landmark-based non-rigid approach to correct inaccuracies. However, this solution is not scalable to the whole genomic database.

We can describe our problem in terms of image registration, whereby the goal is to identify a transformation that maps a moving image to a target reference image. Our task is made particularly challenging by the multimodality of gene expressions with respect to reference Nissl stains and by several artifacts like air bubbles and tears in the brain tissue samples.

In this work, we propose a supervised deep learning framework that efficiently leverages labels provided by a trained expert to accurately register multimodal 2D coronal section images showing gene expression stains. Our approach offers novel contributions in the following aspects:

1. Our model achieves high accuracy and generalizes to new gene expressions and coronal sections. It therefore constitutes a valuable tool for the integration of gene expression brain atlases.
2. By training with a perceptual similarity loss, our model learns to produce smooth deformations without the need any parametric constraint or post-processing stage.

### 1.1. Related Work

There has been some research on registration of Allen Brain datasets. Notably, Xiong et al. (2018) proposed a similarity metric addressing such artifacts and used it to register slices to the reference Nissl volume. Andonian et al. (2019) utilized groupwise registration to create multiple templates that are in turn used for pairwise registration of slices.

Among traditional image registration methods, intensity-based schemes (Klein et al., 2009) such as Symmetric Normalization (SyN) (Avants et al., 2008) represent the most popular approach. They do not require ground truth and rely on maximizing a similarity metric between the reference and registered moving image. These methods usually provide accurate and diffeomorphic predictions. However, they are limited by runtime overhead due to their intrinsically iterative nature, and also require a careful choice of hyperparameters. In particular, in the case of multimodal images like ours, tuning the pre-processing stages and the choice of the similarity metric required several time consuming trial-and-error iterations. In contrast, the model we propose can be easily deployed and used *as-is*, without the need for any tuning.

To address the limitations of traditional intensity-based approaches like SyN, several deep learning solutions have been proposed. Many approaches, such as VoxelMorph (Dalca et al., 2018; Balakrishnan et al., 2019), focused on unsupervised registration of magnetic resonance volumes following a similar approach to intensity-based models. Even though these methods reduced the runtime of the registration process, they cannot yield an improvement in accuracy over intensity-based methods, since they seek to optimize the same loss function (Lee et al., 2019). Furthermore, VoxelMorph maximizes cross-correlation, which is effective on unimodal data like magnetic resonance volumes, but fails on our multimodal images.

Among supervised approaches, RegNet (Sokooti et al., 2017) minimized mean absolute error with respect to a ground truth displacement field without adding any penalty guaranteeing smooth transformations. Moreover, this approach relied on synthetic training data and is therefore necessarily limited to unimodal problems and is therefore not applicable to our data.

Another popular supervised model is SVF-Net (Rohé et al., 2017), which has the advantage of training the model on ground truth transformations derived from region segmentation. The framework is based on training a network to align the boundaries of a pre-defined region of interest, which is not suitable for our use case since the visible brain regions vary across coronal sections.

Finally, while our proposed model learns to predict smooth deformations solely through the usage perceptual loss, previous methods relied either on: (i) parametric approaches like B-splines (de Vos et al., 2017), which restrict the space of possible deformations; (ii) introducing an explicit penalty term in the loss function (Balakrishnan et al., 2019), which further increases the number of hyperparameters; or (iii) integrating a predicted velocity field (Dalca et al., 2018), which requires post-processing steps.

The idea of training a model for image regression with a perceptual loss that uses the features extracted by a pre-trained network was first introduced in Johnson et al. (2016). In that work, the authors tested the approach on style transfer and super-resolution problems and showed that training with this loss produced models that better predict complex features such as texture and sharpness. The intuition behind this work was confirmed by Zhang et al. (2018), which proved that, on a variety of image datasets, the perceptual loss outperforms classical metrics in terms of correlation with human judgement. Perceptual loss has since then been successfully applied to various image generation tasks. To name a few, Huang et al. (2018) improved their results on higher resolutions when working on image-to-image translation, while Li et al. (2020) obtained artifact reduction and structure preservation on image denoising tasks.

Compared to these previous works using perceptual similarity, our approach also relies on the perceptual loss in order to learn to predict outputs that preserve complex visual features of the ground-truth, namely the smoothness of the displacements. However, our approach introduces elements of novelty in that we compute perceptual loss on the components of the displacement vector field rather than on images, and moreover we apply this approach to a new task such as multimodal image registration.

## 2. Materials and Methods

Given a reference image *I*_ref_ and a moving image *I*_mov_, image registration is defined as the problem of finding a transformation ϕ such that the registered image *I*_reg_ = *I*_mov_°ϕ is as similar as possible to the reference *I*_ref_. In the following, we assume that our input consists of a pair of multimodal images $I_{\text{ref}},I_{\text{mov}}\in {\mathbb{R}}^{H\times W\times C}$ (*H*=height, *W*=width, *C*=number of channels), and that the output we want to predict is a transformation represented by an array ϕ∈ℝ^*H*×*W*×2^ such that for every pixel (*x, y*) in *I*_ref_, ϕ(*x, y*)∈ℝ^2^ defines the corresponding position of that pixel in *I*_mov_. Equivalently, one can predict the per-pixel displacement *u*∈ℝ^*H*×*W*×2^ such that *u*(*x, y*) = ϕ(*x, y*)−(*x, y*).

The method we propose is based on supervised learning, so we assume that we have access to training samples (*I*_ref_, *I*_mov_, ϕ) where the ground truth label ϕ is provided by a human expert. These labeled samples are used to train a neural network model as described in the remainder of this section.

All the relevant code can be found at https://github.com/BlueBrain/atlas_alignment.

### 2.1. Network Architecture

Registration methods can be classified based on the family of transformations considered for the predicted deformation $\hat{\phi }$. Our model predicts pixel-wise displacements û, so that it is non-parametric and allows for elastic transformations. This represents a considerable advantage in terms of expressive power in contrast to parametric models, such as affine or thin plate spline methods.

**Figure 1:**
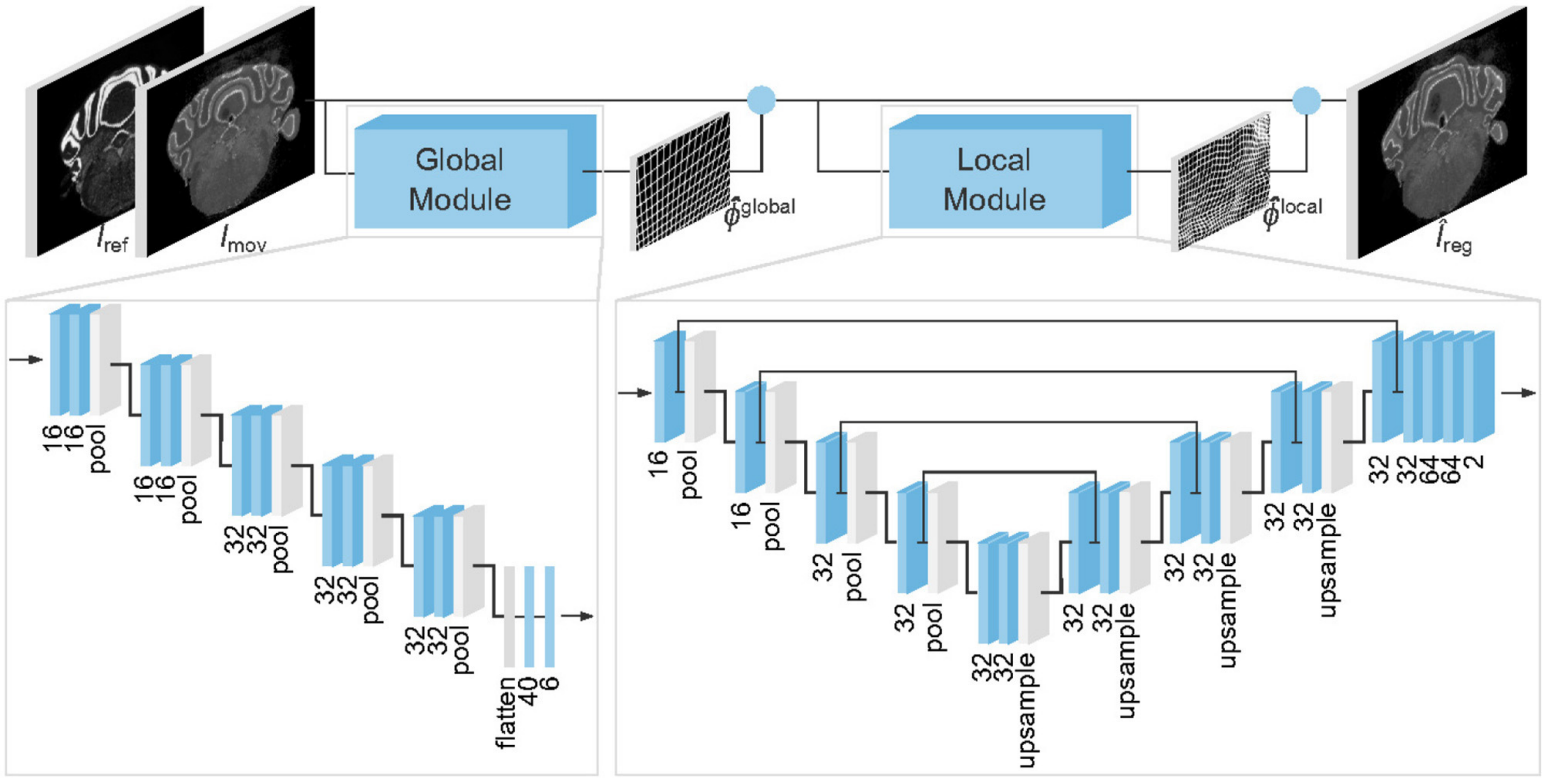
Architecture of the proposed model to register gene expressions *I*_mov_ to Nissl stains *I*_ref_. Blue boxes represent trainable layers. Under all 3 × 3 convolutional layers (3D boxes) and fully connected layers (2D boxes) we display the number of channels and dense units, respectively. Blue circles denote where predicted transformations $\hat{\phi }$ are applied.

Specifically, the architecture of the neural network we propose is shown in Figure 1. Our model consists of two modules, predicting an affine (global) transformation ${\hat{\phi }}^{\text{global}}$ and an elastic (local) deformation ${\hat{\phi }}^{\text{local}}$, respectively. Our final prediction is the composition of the two transformations $\hat{\phi }={\hat{\phi }}^{\text{global}}ₒ{\hat{\phi }}^{\text{local}}$.

Unlike many related works on medical image registration (Sokooti et al., 2017; Yang et al., 2017; Balakrishnan et al., 2018; Dalca et al., 2018), we do not assume that our inputs are pre-centered and rescaled. Consequently, we employ a global alignment module to simplify the registration of the local one.

The architecture of the global and local modules are inspired by the Spatial Transformer Network (Jaderberg et al., 2015) and VoxelMorph (Balakrishnan et al., 2018), respectively.

### 2.2. Loss Function

In the case of multimodal registration, measuring image similarity between reference *I*_ref_ and predicted registration ${Î}_{\text{reg}}=I_{\text{mov}}ₒ\hat{\phi }$ without pre-processing may provide misleading information due to the different appearance of these images. Thanks to our supervised learning framework, we can instead directly compare predictions û and Î_reg_ with ground truths *u* and *I*_reg_, respectively.

We train our model using a loss function composed of three terms

(1) $$\begin{array}{r} L_{\text{tot}}=L_{\text{IE}}+L_{\text{EPE}}+L_{\text{LPIPS}}. \end{array}$$

The loss term *L*_IE_ is an *image error* between the predicted registered image Î_reg_ = *I*_mov_°ϕ and the ground truth *I*_reg_. As the two images have the same modality, pre-processing is unnecessary, and we can simply take

(2) $$\begin{array}{r} L_{\text{IE}}=||I_{\text{reg}}-{Î}_{\text{reg}}|{|}_{2}^{2}. \end{array}$$

The second term *L*_EPE_ is the squared average *endpoint error*, which is commonly used as a metric for optical flow estimation (Zhu et al., 2017). We define this loss as

(3) $$\begin{array}{r} L_{\text{EPE}}={\left(\overset{H}{\underset{x=1}{\sum }}\overset{W}{\underset{y=1}{\sum }}\frac{||u\left(x,y\right)-û\left(x,y\right)|{|}_{2}}{HWT}\right)}^{2}, \end{array}$$

where *T* is a normalizing constant representing the average displacement size computed from training data (in our case, *T*≈20).

Note that *L*_EPE_ is a pixel-wise loss which does not take into account information from neighboring pixels. As a consequence, our model often predicted non-smooth fields $\hat{\phi }$ with a significant number of corrupted pixels, i.e., (*x, y*) where the Jacobian $J_{\hat{\phi }}\left(x,y\right)\in {\mathbb{R}}^{2\times 2}$ has a non-positive determinant. In order to teach the model to predict transformations with smooth texture as the ground truth, we introduce in our total loss *L*_tot_ the loss term *L*_LPIPS_ defined as the *Learned Perceptual Image Patch Similarity Loss* version 0.1 with VGG-lin configuration (see Zhang et al., 2018 for details), which allows us to generate deformations that are perceptually similar to ground truth labels, including smoothness properties. To this end, we view the *x* and *y* components of *u* as two images.

Unlike other traditional metrics, *L*_LPIPS_ not only compares pixel-wise differences but also extracts and compares feature maps using a pre-trained VGG (Simonyan and Zisserman, 2014) network and then computes differences between these deep features. As explained in section 1.1, perceptual similarity is known to be effective in a variety of tasks where complex image features such as texture or sharpness have to be preserved in the predictions. In our case, the two images we compare are the ground truth and the predicted transformation, and the qualitative feature traits we try to preserve by relying on *L*_LPIPS_ consist in the smoothness of the ground truth displacements. Our results, presented and discussed in section 3.2, confirm the validity of this idea.

Finally, inspired by Zhao et al. (2019), we train our model to predict not only how to register *I*_mov_ to *I*_ref_, but also *I*_ref_ to *I*_mov_. We therefore effectively use $L_{\text{EPE}}^{\prime }=L_{\text{EPE}}\left(u,û\right)+L_{\text{EPE}}\left(u^{-1},\hat{u^{-1}}\right)$ and $L_{\text{LPIPS}}^{\prime }=L_{\text{LPIPS}}\left(u,û\right)+L_{\text{LPIPS}}\left(u^{-1},\hat{u^{-1}}\right)$. Given the ground truth *u*, we compute *u*^−1^ numerically using *scattered data interpolation* (SDI) (Crum et al., 2007).

To minimize the loss function *L*_tot_ we apply the RMSProp (Root Mean Square Propagation) optimizer (Tieleman and Hinton, 2012) which has a learning rate η = 10^−3^ and a forgetting factor γ = 0.9. Additionally, the batch size is set to 4 due to GPU memory limitations.

### 2.3. Data Augmentation

To improve the generalization performance of our model, we generate synthetic samples of the form $\left(I_{\text{ref}},I_{\text{mov}}^{\prime },\phi^{\prime }\right)$ from each training sample (*I*_ref_, *I*_mov_, ϕ).

A first class of augmentations generates $I_{mov}^{\prime }$ from *I*_*mov*_ by applying random blurring, brightness perturbation, and other image processing techniques. These augmentations help improve the accuracy of predictions on images having different perceptual appearance, and generalize to gene expressions not present in the training set. Note that for these augmentations ϕ′ = ϕ.

Another class of augmentations consists of geometric transformations, affecting both *I*_mov_ and ϕ. These are particularly relevant for our application, since our focus is on predicting elastic deformations. First, control points are sampled on the edges of a brain section, and random displacements are generated for each of these points. Interpolating these displacement vectors with radial basis functions yields a smooth transformation ψ defined over the whole *I*_mov_. We obtain a synthetic sample by considering $I_{\text{mov}}^{\prime }=I_{\text{mov}}ₒ\psi$ and ϕ′ = ϕ°ψ^−1^.

### 2.4. Dataset and Evaluation Metrics

The reference Nissl stain volume of the AMBA comprises 528 coronal sections. Typically 8 markers per specimen were assayed, yielding approximately 60 coronal sections per gene expression. Our goal is to register the moving gene expression *I*_mov_ to the reference Nissl slice *I*_ref_.

In order to train and evaluate our model, we selected 277 section pairs from the Nissl atlas and 7 different gene atlases for calbindin (CALB1), calretinin (CALB2), cholecystokinin (CCK), neuropeptide Y (NPY), parvalbumin (PVALB), somatostatin (SST), and vasointestinal peptide (VIP). Even though all the gene expressions were pre-aligned using the affine registration module provided by the Allen Brain API, significant misalignments were still present. The original sections have various resolutions, so we had to rescale the images in order to be able to run our model, which assumes all moving and reference inputs to have the same shape. We therefore downscaled all slices to a fixed 320 × 456 pixels resolution, which corresponds to a 25 μm sampling distance that is the same value of the slices thickness in the Nissl atlas, in order to have a uniform resolution across the three axes. Finally, all images were converted to grayscale.

We collected ground truth labels from a human expert provided with a manual landmark-based non-rigid registration tool that we designed to export the deformation field and registered image. This annotation tool is named label-tool and is part of our open-source Python package. On average the expert used 27.7 keypoint pairs (with a standard deviation of 10.7) to register a sample. However, the number required keypoints significantly depends on the gene expression and on the coronal section location, as shown in Table 1. This provides a further argument in favor of our supervised learning approach, which exports the whole deformation field provided by a human expert and does not constrain the annotator to a fixed number of control points, unlike the case of parameteric models such as de Vos et al. (2017).

**Table 1:** Average number of keypoint pairs used by the annotation expert (per gene and coronal section group).

**Gene**	**1–176**	**177–352**	**353–528**
CALB1	19.1	36.3	40.8
CALB2	18.7	36.9	39.4
CCK	18.6	30.4	35.1
NPY	19.4	27.4	27.0
PVALB	17.9	24.4	37.3
SST	17.7	29.6	26.0
VIP	19.6	31.2	33.4

**Figure 2:**
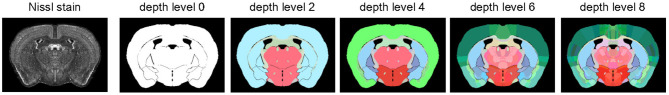
Hierarchical segmentation of a Nissl stain (coronal section 250) used to compute Dice score to evaluate our model. Level 0 only distinguishes background from foreground, while deeper levels define an increasing number of brain subregions.

To measure the performance of our model, we considered the hierarchical segmentation maps provided by the AMBA to compute the average Dice score (Dice, 1945) (using weights proportional to the number of pixels of each segmentation class) at different levels, as shown in Figure 2. We performed this comparison in the moving space by warping the ground truth segmentation by ϕ^−1^ and ${\hat{\phi }}^{-1}$ (both computed numerically). As a benchmark, we used an affine model and SyN as implemented in the Advanced Normalization Tools (ANTs) software package (Avants et al., 2011). We opted for mutual information as a similarity metric to handle multimodality.

## 3. Results

### 3.1. Quantitative Analysis

We evaluated the performance of our model on two different experiments. In the first experiment, we applied an 80:20 train-test split using a stratified partitioning scheme based on the different genes and on the section locations on the anterior-posterior axis.

As indicated in Table 2, our model outperforms both the affine model and SyN with respect to Dice score. The improvement over SyN is marginal for level 0, which corresponds to a background-foreground segmentation as shown in Figure 2. However, our model's relative advantage increases as we consider more regions. Indeed, aligning complex brain structures in multimodal images is a harder task for intensity-based models. Table 2 shows that our model tends to predict smooth transformations with only 0.11% of corrupted pixels, mostly occurring at image borders. This is particularly noteworthy since the smoothness emerges naturally from training with the loss function defined in section 2.2.

**Table 2:** Summary of results on an 80:20 train-test stratified data split (mean and standard deviation in percentage).

**Model**	**Dice-0**	**Dice-2**	**Dice-4**	**Dice-6**	**Dice-8**	**$|J_{\hat{\phi }}|\leq 0$**
Ours	**94.2** ±**4.0**	**84.4** ±**6.9**	**80.6** ±**7.9**	**68.0** ±**13.3**	**55.2** ±**11.9**	0.11 ± 0.17
SyN	94.1 ± 4.2	83.9 ± 7.6	79.8 ± 8.8	66.1 ± 13.9	52.3 ± 12.5	0.01 ± 0.02
Affine	91.4 ± 5.9	79.9 ± 10.0	75.5 ± 11.0	61.2 ± 17.7	46.8 ± 15.8	0.00 ± 0.00

*Bold values indicate the highest (= best) Dice score in the various experiments*.

In the second experiment, we studied how our model generalizes to new genes by training on slices of 6 genes and evaluating performances on the remaining holdout gene. Results in terms of Dice-8 score, where difference between models is more visible, are reported in Table 3. Even in this more difficult scenario, where slices of the holdout gene are never shown to the model during the training phase, our network achieves higher scores than SyN on all but one gene. The overall results of this second experiment confirm that our model generalizes to new genes and is therefore suitable for registering and leveraging multimodal gene atlases.

**Table 3:** Summary of results on a gene-holdout split (Dice-8, mean, and standard deviation in percentage).

**Model**	**CALB1**	**CALB2**	**CCK**	**NPY**	**PVALB**	**SST**	**VIP**
Ours	**48.8** ±**9.5**	**55.3** ±**12.3**	**54.5** ±**13.0**	**44.4** ±**12.5**	56.0 ± 13.6	**60.3** ±**12.4**	**58.4** ±**13.3**
SyN	46.9 ± 10.9	54.6 ± 12.9	50.7 ± 14.3	41.7 ± 15.4	**58.5** ±**10.6**	57.0 ± 12.5	55.6 ± 11.5
Affine	40.5 ± 15.8	51.5 ± 14.3	46.7 ± 12.0	36.6 ± 15.5	53.0 ± 12.1	54.3 ± 13.4	52.5 ± 14.3

*Bold values indicate the highest (= best) Dice score in the various experiments*.

Finally, running on an Intel Core i7-4770 CPU, registering a sample takes either ~3 s or ~0.2 s using SyN or our model, respectively. On an NVIDIA Tesla V100 GPU, the runtime of our model is further reduced to ~0.009 s (the ANTs package does not provide GPU implementations of SyN). These results demonstrate that our approach is also competitive in terms of runtime.

### 3.2. Qualitative Analysis

**Figure 3:**
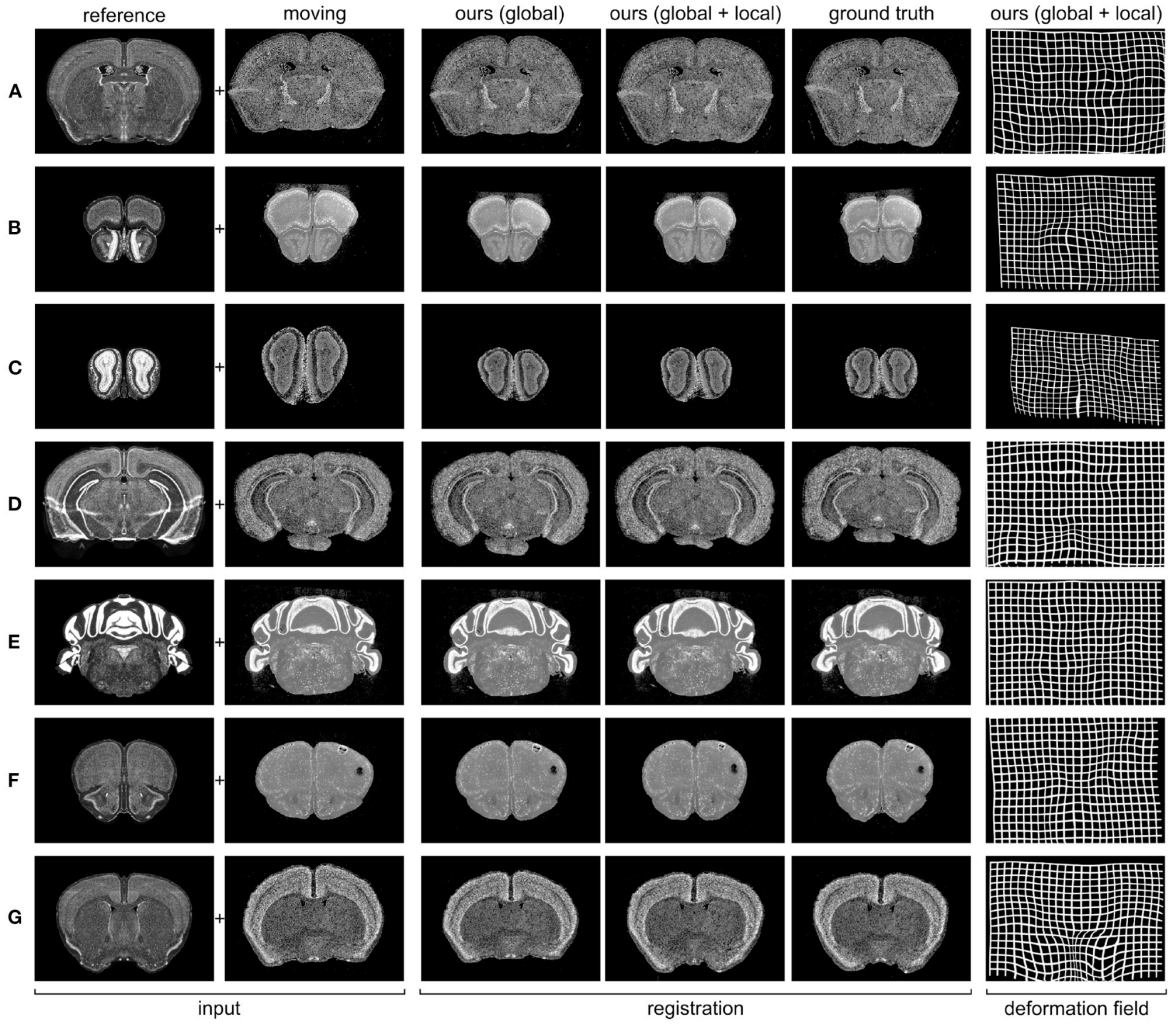
Predicted registrations on slices from the 7 different gene expression atlases used in our experiments (see section 2.4 for details). **(A)** PVALB gene, section 236; **(B)** CALB1 gene, section 100; **(C)** NPY gene, section 52; **(D)** SST gene, section 328; **(E)** CALB2 gene, section 451; **(F)** VIP gene, section 129; **(G)** CCK gene, section 190.

A qualitative analysis of the predictions of our model is shown in Figure 3. Our global module provides a first affine transformation that rescales and centers the moving image. The need and the efficacy of this module are particularly visible in the case of samples (Figures 3B,C), where the global module significantly rescales and shifts the input gene expression. The local module then applies an elastic deformation that accurately aligns the gene expression to the reference Nissl stain.

**Figure 4:**
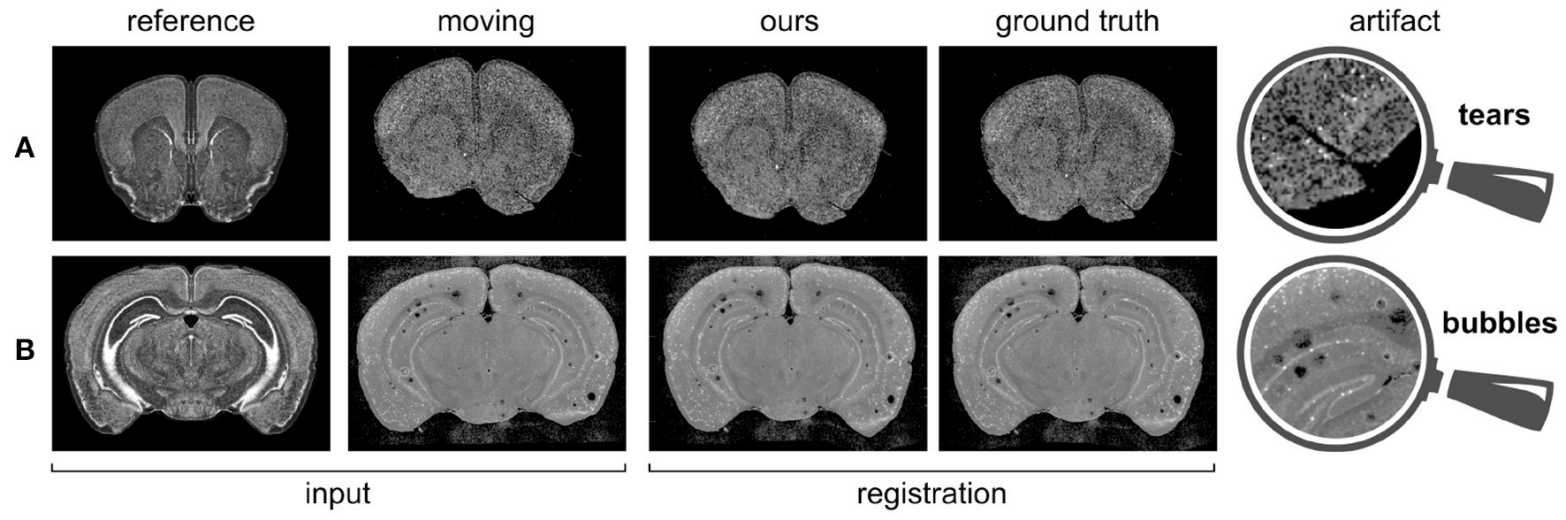
Gene expressions containing artifacts, and corresponding predicted registrations. **(A)** PVALB gene, section 160; **(B)** VIP gene, section 316.

We already mentioned in section 1 that our registration task is made particularly challenging by the presence of tears and air bubbles in the gene expression stains. In Figure 4, we demonstrate the stability of our approach by showing examples of gene expression slices including these kinds of artifacts together with the ground truth and predicted registrations.

**Figure 5:**
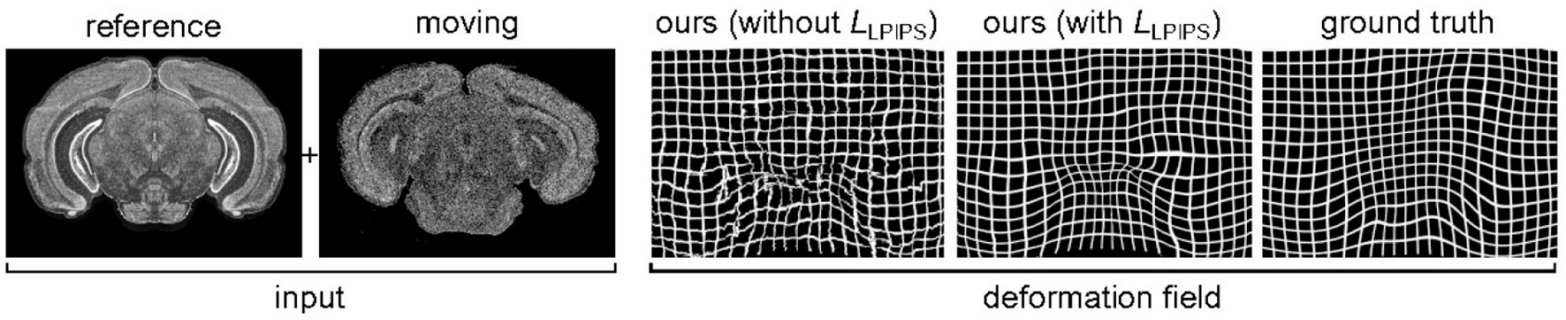
Influence of the loss function on the smoothness of the predicted deformation, for SST gene, section 352. If we use a loss without perceptual similarity, ~3% of pixels are corrupted. By introducing the *L*_LPIPS_ term, this is reduced to ~0.1%.

As explained previously in section 2.2, the smoothness of the predicted deformation field $\hat{\phi }$ can be entirely ascribed to our choice of loss function. Figure 5 illustrates how results vary depending on whether or not *L*_tot_ includes the perceptual similarity term *L*_LPIPS_. Notice that, without this term, the model produces a significant number of corrupted pixels.

**Figure 6:**
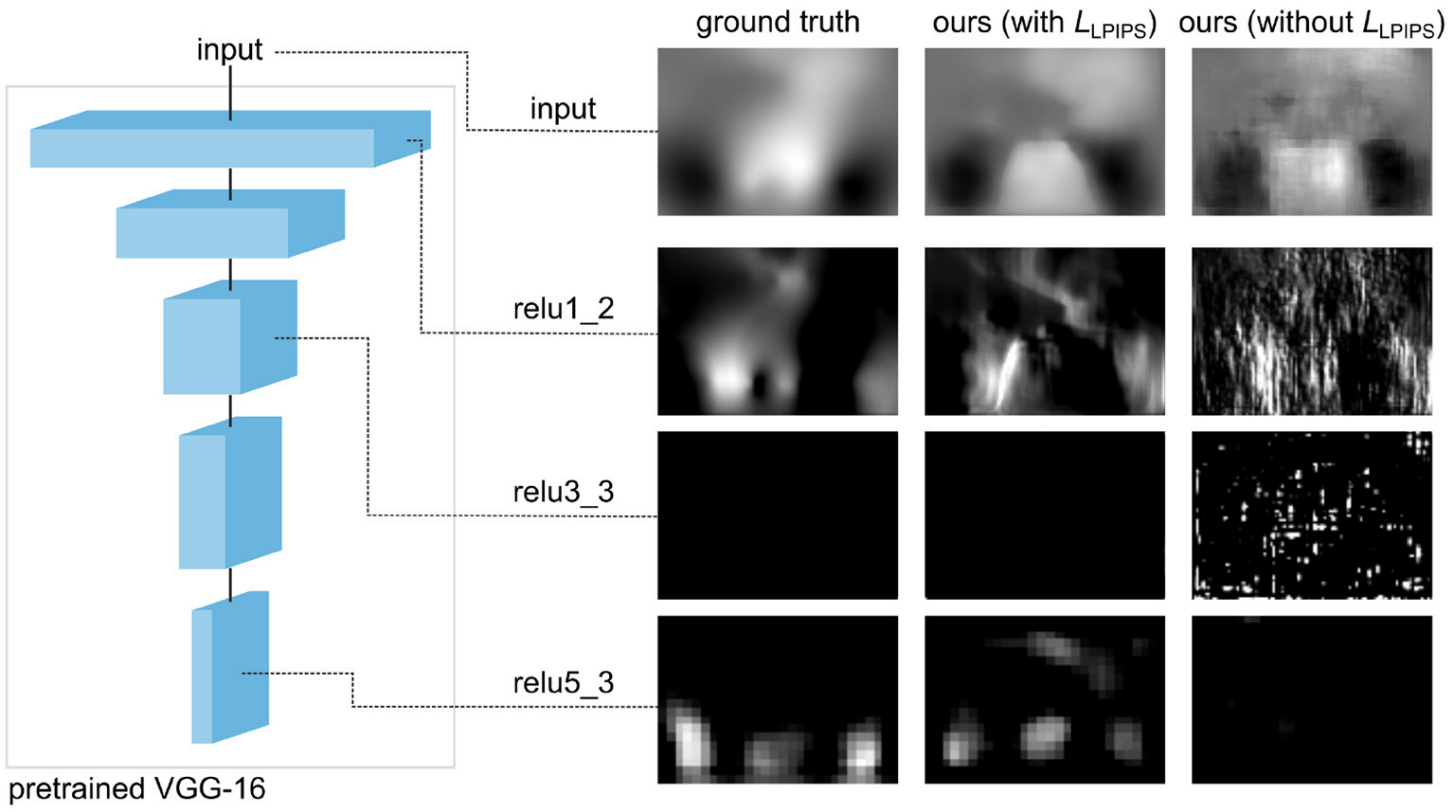
Activations of the pre-trained network used to compute *L*_LPIPS_ on the *y* component of the transformation *u* for the coronal section 352 of SST gene (same as in Figure 5). The deep features of the non-smooth predicted û obtained by training without *L*_*LPIPS*_ significantly differ from those of smooth transformations corresponding to the ground truth *u* and predicted û obtained by training with *L*_LPIPS_.

Further insight with respect to these results is provided in Figure 6, where we can observe some of the feature maps used to compute *L*_LPIPS_. As previously described, these deep features are the internal activations of a pre-trained VGG network. The similar, smooth appearance of the ground truth *u* and predicted transformation û obtained by training with *L*_LPIPS_ is well-captured by these activations, which look significantly different for the non-smooth predicted transformation û we obtained when training without the *L*_LPIPS_ term. These observations help justify the importance of using the perceptual loss in our framework to produce smooth results.

Interestingly, if we evaluate the predicted transformations shown in Figure 6 using *L*_EPE_, the prediction obtained by training with the perceptual loss (*L*_EPE_ = 6.83) seems to be worse than the one obtained without it (*L*_EPE_ = 6.03). This strongly contrasts with the fact that this latter looks smooth and qualitatively similar to the ground truth, while the other prediction clearly includes a large number of artifacts. However, if we evaluate the same transformations using *L*_LPIPS_ we reach the opposite conclusions, as the prediction obtained by training with the perceptual loss (*L*_LPIPS_ = 0.30) appears to be better than the one obtained without it (*L*_LPIPS_ = 0.53). These results are consistent with Zhang et al. (2018), where perceptual similarity is shown to strongly correlate with human perception, unlike other traditional metrics.

## 4. Discussion

In this paper, we presented a supervised deep learning model with perceptual similarity for the 2D registration of gene expressions to Nissl stains of the Allen Mouse Brain Atlas. The main novelty of our method lies in its unique non-parametric approach which allows the prediction of smooth deformations by exclusively relying on a perceptual loss function. In contrast to this, previous works had to resort to using parametric methods, extra penalty terms with hyperparameters requiring careful tuning, or post-processing steps.

By testing on two different experiments, we showed that the proposed approach produces accurate predictions that generalize well to unseen gene expressions and coronal sections. This is particularly significant given the high variability of shape and appearance across stains and sections, as shown in Figure 3. We benchmarked our results against the state-of-the-art method SyN, and our results showed that our model is significantly faster and it also achieves higher accuracy in almost all cases.

Our qualitative analysis shows that our model is able to predict deformation fields that are very close to the ground truth annotations provided by a human expert, even in case of slices affected by artifacts such as air bubbles and tears. Indeed, during the training phase, our model is presented with samples including various kinds of anomalies, and therefore learns how to predict a deformation field in a correct way, as opposed to intensity-based approaches.

Our framework has therefore proven capable of enabling the neuroscience community to leverage large-scale complex brain-derived datasets, with a significant scientific impact in terms of acceleration and accuracy improvement.

We identify three drawbacks of the presented approach. Firstly, it assumes that we have access to expert labels. Manual registration with any annotation tool is a difficult task and the resulting ground truth deformation might vary from one expert to another. The second shortcoming is that a generalization of our approach to 3D registration is not straightforward. This is due to the fact that perceptual loss is computed on images rather than volumes. Lastly, the training of our neural network represents the most time consuming stage of the pipeline. This is a common problem of many deep learning models and it should not be completely overshadowed by fast inference.

The future research direction is to apply our approach to new datasets. One specific example is to investigate sagittal sections. In general, the most promising applications are in the registration of multimodal datasets where using traditional approaches might lead to inaccurate results.

## Data Availability Statement

The datasets presented in this study can be found in online repositories. The names of the repository/repositories and accession number(s) can be found in the article/supplementary material.

## Author Contributions

JK and FC conceived and designed the method. JK, CE, HL, and DR collected the data and including the annotations. All authors contributed to interpreting the results and writing the paper, approved the final version, and agreed to be accountable for all aspects of the work.

## Conflict of Interest

The authors declare that the research was conducted in the absence of any commercial or financial relationships that could be construed as a potential conflict of interest.

## Publisher's Note

All claims expressed in this article are solely those of the authors and do not necessarily represent those of their affiliated organizations, or those of the publisher, the editors and the reviewers. Any product that may be evaluated in this article, or claim that may be made by its manufacturer, is not guaranteed or endorsed by the publisher.
